# Comparison of Renal Growth, Proteinuria and Graft Survival between Recipients of Pediatric and Adult Cadaveric Kidney Transplants

**Published:** 2017-05-01

**Authors:** A. Basiri, S. Zare, N. Simforoosh, A. Tabibi, M. H. Shakibi

**Affiliations:** 1 Urology and Nephrology Research Center, Shahid Beheshti University of Medical Sciences, Shahid Labbafinejad Medical Center, Tehran, Iran; 2 Shahid Sadoughi University of Medical Sciences, Yazd, Iran; 3 Shahid Labbafinejad Medical Center, Tehran, Iran

**Keywords:** Tissue donors, Donor selection, Kidney, Survival, Graft survival, Proteinuria, Postoperative complications, Transplant recipients, Pediatric

## Abstract

**Background:**

The shortage of cadaveric kidney donors has prompted transplant teams to expanding the donor selection criteria. The usage of pediatric cadaveric kidneys is one of those expanded criteria. But the main concern is the probability of hyperfiltration syndrome due to small renal mass of pediatric donors.

**Objective:**

To compare the graft and patient survivals, post-transplantation complications, rate and severity of proteinuria secondary to hyperfiltration injury and the kidney growth of recipients who underwent transplantation from adult (group 1) and pediatric deceased donors (group 2).

**Methods:**

In this historical cohort study, each group contains 36 patients. Outcome measures included patient and graft survivals, quality of graft function as assessed by serum creatinine (SCr) and estimated GFR (eGFR), surgical complications, proteinuria that was detected by routine urinalysis and then confirmed by a 24-h urine protein >150 mg, blood pressure, and kidney length and volume measured by early and follow-up ultrasonography.

**Results:**

The mean donor age in groups 1 and 2 was 36 and 6.5 years, respectively. 9 (25%) kidneys taken from pediatric donors (group 2) were offered *en-bloc*. The mean follow-up was 28 month. The two groups were not significantly different in terms of the incidence of DGF, rate of acute rejection, 1-year graft survival, SCr and eGFR levels, rates of surgical complications requiring surgical interventions, development of proteinuria, and rate of post-transplantation rise in blood pressure. The mean±SD kidney length within 24 hours of transplantation was significantly higher in group 1 compared to group 2 recipients (112±14 *vs*. 75±12 mm; p=0.001), but the rate of increase in kidney length in group 2 was significantly higher than that in group 1 recipients (43±4 *vs*. 10±2 mm; p=0.002) during the follow-up period. 80% of the increase in the kidney size was observed during the first 12 months of surgery; another 20% happened between 12 and 18 months.

**Conclusion:**

We found that obligatory and compensatory growth of pediatric kidney donors can overcome the concern of hyperfiltration syndrome and that the outcome is the same as adult donors.

## INTRODUCTION

The disparity between the number of patients with end-stage renal disease (ESRD) on the kidney transplant waiting list and the availability of deceased donor organs continues to grow [1]. The shortage of cadaveric donors for kidney transplantation has encouraged physicians to expand the donor selection criteria. Pediatric cadaveric kidney is one such expanded criteria [2, 3]. Challenges over functional capability, technical problems, and early graft dysfunction are advocated as limitations of transplanting pediatric kidneys to adult recipients in some centers [4]. The lower graft survival of pediatric kidney grafts, due to the technical complications and hyperfiltration injury, is challenging [5, 6]. Several studies have indicated that the outcomes of transplantation, using pediatric donors, are not favorable when compared with results of adult donors [7, 8]. First proposed in 1972, one possible solution to prevent the complications that may occur with using solitary small pediatric kidneys, is *en-bloc* transplantation [9]. However, when the initial complications decrease, several studies have suggested that pediatric kidneys would be considered excellent rather than marginal, for transplantation to adults [10, 11]. Data regarding long-term function and single or *en-bloc* transplantation of small pediatric kidneys are diverging. In this study, we compared the renal growth, proteinuria, and graft function of recipients who underwent transplantation from adult (group 1) or pediatric deceased donors (group 2).

## MATERIALS AND METHODS

In this historical cohort study, we reviewed the hospital records and follow-up data of adult (group 1) and pediatric (group 2) cadaveric kidney donors from June 2006 to January 2013. Each group contains 36 patients. Outcome measures included patient and graft survivals, quality of graft function as assessed by serum creatinine (SCr) and estimated GFR (eGFR), surgical complications, proteinuria detected by routine urinalysis and confirmed by a 24-h urine protein >150 mg, blood pressure, and kidney growth measured by ultrasonography. Those with significant proteinuria before transplantation was excluded from the study. Proteinuria of <150 mg/24 hr urine was considered “normal,” 150–300 mg/24 hr “micro-proteinuria,” 300–1000 “grade 1,” 1000–3500 “grade 2,” and >3500 was considered “grade 3” or “nephrotic range.” *En-bloc* kidney transplantations were offered from pediatric cadaveric donors with at least one of the following criteria: age <5 years, weight <15 kg, or kidney length <8 cm, which were transplanted to adult recipients. The main immunosuppressive agents were cyclosporine, prednisolone, and mycophenolate mofetil. Acute rejection episodes were treated by pulse steroid and antilymphocyte globulin (ATG). The patients were followed for 18–72 (mean: 28) months, equal for the two groups. GFR was estimated based on the updated 2005 Modification of Diet in Renal Disease (MDRD) study equation [12]:

GFR = 175 × (standardized SCr)^−^^1.154 ^× (age)^−^^0.203^ × 0.742 (if the subject is female)

expressed as mL/min/1.73 m^2^. Ultrasonographic measurement of the cranio-caudal length of the transplanted grafts was repeated and compared with the measurement performed within 24 hours post-transplantation. Volume (length × width × thickness × π/6) of the *en-bloc* kidney *vs*. single pediatric kidney and adult-to-adult transplants were compared. In recipients with two *en-bloc* grafts, the volumes of the two kidneys were added.

Statistical Analysis

SPSS ver 16 (SPSS Inc., Chicago, IL, USA) was used for statistical analysis. All univariate comparisons were unpaired; all tests of significance were 2-tailed. For comparisons, analysis of variance was used. Linear regression analysis was used to assess the association between the maximal cranio-caudal length of the kidneys. An additional Cox model was used for renal size parameters. *Student’s* *t* test was used for univariate analysis. Categorical data were compared by χ^2^ test. A p value <0.05 was considered statistically significant.

## RESULTS

**Figure 1:**
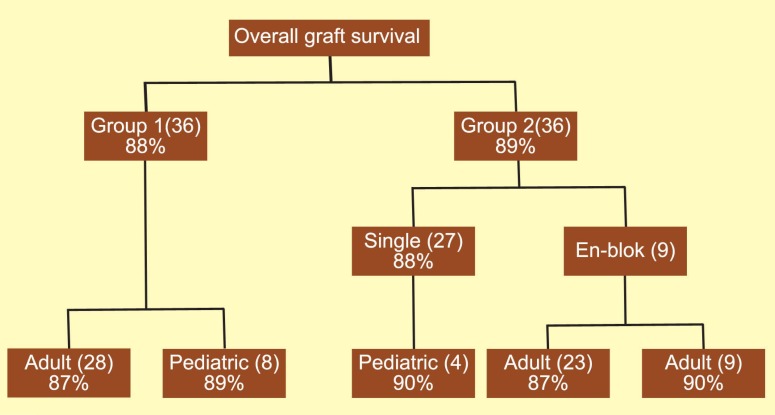
Overall graft survival in adult (group 1) and pediatric (group 2) cadaveric kidney recipients

The mean donor age in groups 1 and 2 were 36 (range: 15–65) years and 6.5 (range: 2.5–15) years, respectively. The pediatric donors included 12 aged ≤5 years, 16 aged 6–10, and 8 aged 11–15 years. The mean±SD age of pediatric recipients was 11±3 (range: 3.5–14) years. Nine (25%) kidneys from pediatric donors (group 2) were offered *en-bloc*. The mean follow-up period was 28 (range: 18–72) months. The underlying diseases of pediatric recipients included obstructive uropathy, hypoplastic/dysplastic kidney disease, reflux nephropathy, and glumerolonephritis. There were no significant differences in the incidence of DGF between the two groups (21% *vs*. 19%) (p=0.62). Group 2 had a slightly higher incidence of acute rejection compared to group 1 (12% *vs*. 8%), but the difference was not significant (p=0.57). One-year graft survival was similar in both groups (90% and 92%). The overall patient and graft survival was, respectively, 91% and 88% in group 1, and 90% and 89% in group 2. Twenty-eight (78%) kidneys of group 1 were transplanted as a single unit to adult recipients; in group 2, 23 (64%) single kidney units were transplanted to adults. An interesting point was the similar overall graft survival (87%) in these 2 groups (Fig 1). 

**Figure 2:**
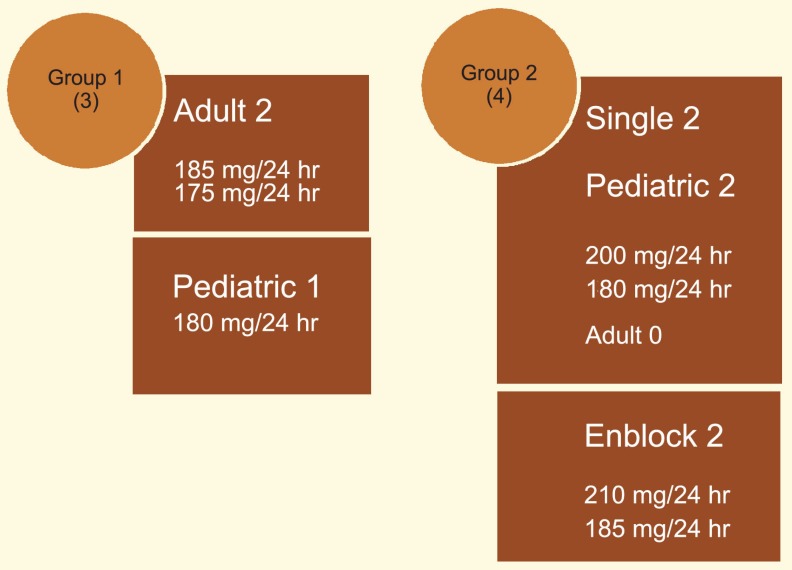
Composition of studied groups

SCr and eGFR were not significantly different between the two groups (1.28 *vs*. 1.31 mg/dL, and 87 *vs*. 88 mL/min, respectively) (p=0.20). The incidences of surgical complications were comparable between two groups (13.8% *vs*. 16.6%) (p=0.42) (Table 1). Development and severity of proteinuria was not different between the two groups (8.3% *vs*. 11%) (p=0.12) (Fig 2). Proteinuria occurred between 6 and 12 months post-operation in all seven cases. In group 1, two patients with proteinuria observed in adult recipients; another one occurred in pediatric recipients. In group 2, proteinuria was detected in two recipients of single kidneys—both were pediatrics, not adult. Other two recipients with proteinuria in group 2 received *en-bloc* kidneys. Proteinuria was <250 mg/24 hr urine volume in all patients. The 80% of the kidney size increase was observed during the first 12 months after the surgery; the remaining 20% occurred between 12 and 18 months (Table 2). The mean arterial blood pressure before and after transplantation was 115 and 120 mm Hg in group 1, and 100 and 105 mm Hg in group 2 patients. The rate of post-transplantation blood pressure rise was not significantly different between the two groups (p=0.2).

**Table 1:** Surgical complications observed in study groups

Complications	Group 1		Group 2	
	To adult	To pediatrics	*En-bloc*	Single
Renal vein thrombosis	—	1 (early nephrectomy)	—	2 (graft loss)
Renal arterial thrombosis	1 (graft loss)	—	—	—
Significant lymphocele	1 (percutaneous drainage)	—	1 (percutaneous drainage)	1
Ureteral fistula	1 (surgical repair)	—	—	1 (surgical repair)
Ureteral stenosis	1 (surgical repair)	—	—	1 (surgical repair)

**Table 2:** Renal length and volume of adult (group 1) and pediatric (group 2) cadaveric donors at early post-operative and follow-up periods. Figures are mean and mean±SD

Mean kidney length (mm) and volume (mL)	Early post-operative	6 m	12 m	18 m	End of follow up	p value
Group 1						
Kidney length	112±14	116±12	120±15	121±14	122±15	0.1
Kidney volume	175.2	182.3	191.2	194.5	196.2	0.3
Group 2						
Single						
Kidney length	75±12	94±15	112±16	116±15	118±15	0.04
Kidney volume	136.2	170.3	185.2	199.4	204.1	0.002
*En-bloc*						
Kidney length	113±16	122±15	135±12	136±14	138±21	0.03
Kidney volume	158.6	210.2	222.4	235.3	243.1	0.003

## DISCUSSION

The current demand of donor kidneys for transplantation far outstrips the growing supply of cadaveric kidneys. As a result, organs from “suboptimal” donors, such as those older than 55 years and younger than 5 years, are being used with increasing frequency to compensate the donor pool [13]. Almost 40 years ago, animal studies suggest that the renal tubular architecture is mature at birth and that growth is associated only with an increase in GFR [14]. Thereafter, several studies have shown that compensatory hypertrophy occurs rapidly within weeks after transplantation into a much larger recipient. It is well confirmed then that a technically successful transplantation from a very young donor can be expected to maintain electrolyte and fluid homeostasis and that functional reserve should increase with post-transplantation hypertrophy [15]. Although most clinical studies have confirmed this finding, challenge still exists that a smaller kidney must suffer the trauma of the transplantation surgery, donor harvest, storage, and possible rejection episodes prior to the complete growth. The use of pediatric cadaveric donors under five years can result in a significant rate of technical problems; main challenges are a probable risk of vascular thrombosis, suboptimal nephron mass, common rejection episodes, rate of graft survival, hyperfiltration injury, and complexity in adjustment of immunosuppression [16].

In our study, the incidence rates of complications requiring surgical intervention (urinary leakage, ureteral stenosis, lymphocele, vascular thrombosis) were similar between the two studied groups (13.8% *vs*. 16.6%). Vascular complications were detected in 5.5% of cases of each group.

In reports of transplantation using single or *en-bloc* small pediatric donors (15–20 kg or <2 years of age), the rate of thrombosis ranged from 2.5% to 12.5%. Graft thrombosis has been correlated to hypoperfusion or hypotension, torsion of vascular pedicle, small vessel size, thrombus formation within the blind end of aorta and/or vena cava with subsequent propagation, hypercoagulable state, and acute rejection. The outcome also depends on the surgical technique used [17]. Splitting *en-bloc* kidneys and transplanting a solitary pediatric kidney into one recipient could double the number of kidney transplants from one donor, but a marginal results may be expected. The graft survival of *en-bloc* kidneys has been shown to be similar to that of adult cadaveric donors and probably even live-donor kidneys [1-10]. Dharnidharka, *et al*, reported that pediatric *en-bloc* kidney transplantation has greater long-term graft survival compared to single kidney transplants—71% *vs.* 63%, respectively [3]. Pelletier, *et al*, compared graft and patient outcomes of *en-bloc* (n=1301) and single kidneys (n=1175) of small pediatric cadaveric donors under 21 kg, and found a five-year graft survival of 72.7% and 54.8%, respectively [1].

In our study, one-year graft survival was similar in both studied groups (90% and 92%). Overall patient and graft survival was 91% and 88% in group 1, and 90% and 89% in group 2, respectively. 

Although handling of small pediatric single unit renal vessels is challenging, some researchers suggest that use of single kidneys (rather than *en-bloc*) reduces the probability of vascular torsion, and that applying a wide aortic Carrel patch reduces the risk of anastomotic stenosis [18]. In contrast, some researchers show that *en-bloc* technique results in a better outcome in a higher percentage of recipients [19]. United network for organ sharing (UNOS) data from 1987 to 2003 suspected that the graft survival of *en-bloc* kidneys at 1, 3, and 5 years was superior (85%, 76%, and 71%) to that of single-kidney units (81%, 68%, and 63%) from donors aged under 5 years (p<0.001) [3]. Scientific Registry of Transplant Recipients data from 1993 through 2002 were remarkable for a 78% higher risk for graft loss among recipients of single kidneys compared with *en-bloc* transplants from donors who weighed less than 21 kg [1]. While lower donor weight independently promotes a higher risk of graft dysfunction, some authors indicated that there was no significant difference in long-term graft survival between kidneys from donors less than 10 kg transplanted *en-bloc* compared to solitary kidney transplants from donors weighing 15–21 kg, as shown in our study. Although the current opinion of using *en-bloc* *vs*. single pediatric kidney is usually based on the donor age of 5 years or body weight of 20 kg in the literature, it seems that the most important determinant factor is individual transplant surgeon decision during the surgery [20]. 

We offered *en-bloc* kidney transplantations from pediatric cadaveric donors with at least one of these criteria: age <5 years, weight <15 kg, and kidney length <8 cm, which were transplanted to adult recipients. Overall graft survival was not different between single and *en-bloc* groups (88% and 90%, respectively).

There are a few concerns about compensatory hypertrophy and the probability of substantial hyperfiltration injury secondary to increase nephron mass and GFR. In our study, the rate of renal growth in pediatric donors was significantly greater than adult donors; this growth mostly acquired during the first 12 months of transplantation. 

A prospective study reports that pediatric kidneys transplanted to adults have a subsequent potential for growth especially during the first year, and propose that this increase of nephron size and renal mass may be subsequently combine with 20% improvement in renal function. The acceptable transplant function at 1-year without signs of hyperfiltration syndrome such as proteinuria and hypertension can subject a positive signal for good long-term function of these pediatric kidney transplants [11]. Development and severity of proteinuria in our study was not different between the two groups (8.3% *vs*. 11%) (p=0.12). Proteinuria in all of 7 cases occurred between 6 and 12 months post-transplantation. In group 1, two cases of proteinuria were observed in adult recipients; another one occurred in a pediatric recipient. In group 2, proteinuria was detected in two cases of single offered kidney donors; these two recipients were pediatrics, not adult. Other two cases of proteinuria in group 2 were in *en-bloc* offered kidney donors. Proteinuria was <250 mg/24 hr urine volume in all patients. We found that the mean arterial blood pressure before and after transplantation was 115 and 120 mm Hg in group 1, and 100 and 105 mm Hg in group 2. The rate of post-transplant blood pressure rise was not significantly different between the two groups (p=0.4).

Nghiem, *et al*, observed in 21 transplantations of *en-bloc* pediatric kidneys to adults a 2–3-fold increase in size and acceptable function of the kidneys during the first year, without exhibition of proteinuria or hypertension [21]. 

Borboroglu, *et al*, showed in 15 single kidney donors, excellent graft function and no sign of hyperfiltration injury if donor weight was >14 kg and the length of the donor kidney was ≥6 cm [22].

In conclusion, our study showed that obligatory and compensatory growth of pediatric kidney donors can overcome the concern of hyperfiltration syndrome. Median-term outcomes and complications of single and *en-bloc* kidney transplantation from pediatric donors are acceptable and the same as those from older donors. However, better assessment of functional and hemodynamic adaptation of small pediatric kidneys transplanted in adult recipients and subsequent hyperfiltration effects, requires further study on more cases with long-term follow-up.
